# Utterance-final position and pitch marking aid word learning in school-age children

**DOI:** 10.1098/rsos.161035

**Published:** 2017-08-16

**Authors:** Piera Filippi, Sabine Laaha, W. Tecumseh Fitch

**Affiliations:** 1 Department of Cognitive Biology, University of Vienna, Vienna, Austria; 2 Department of Linguistics, University of Vienna, Vienna, Austria

**Keywords:** word learning, language acquisition, prosody, memory, recency, cross-situational learning

## Abstract

We investigated the effects of word order and prosody on word learning in school-age children. Third graders viewed photographs belonging to one of three semantic categories while hearing four-word nonsense utterances containing a target word. In the *control* condition, all words had the same pitch and, across trials, the position of the target word was varied systematically within each utterance. The only cue to word–meaning mapping was the co-occurrence of target words and referents. This cue was present in all conditions. In the *Utterance-final* condition, the target word always occurred in utterance-final position, and at the same fundamental frequency as all the other words of the utterance. In the *Pitch peak* condition, the position of the target word was varied systematically within each utterance across trials, and produced with pitch contrasts typical of infant-directed speech (IDS). In the *Pitch peak + Utterance-final* condition, the target word always occurred in utterance-final position, and was marked with a pitch contrast typical of IDS. Word learning occurred in all conditions except the control condition. Moreover, learning performance was significantly higher than that observed with simple co-occurrence (*control* condition) only for the *Pitch peak + Utterance-final* condition. We conclude that, for school-age children, the combination of words' utterance-final alignment and pitch enhancement boosts word learning.

## 1. Introduction

A central issue in the study of language acquisition concerns the perceptual and memory constraints that human learners are subjected to as anchor points for word learning [1,2]. A number of experimental studies show that language learners are able to associate the sound of a word with its referent, at the moment the novel word is first encountered, or given repeated unambiguous pairings in a single session [3,4]. However, word learning may go beyond the process of learning one label associated with one object [5]. In fact, language learners are typically exposed to many words and many potential referents, and need to use specific cues as to which sounds refer to which referents in the surrounding visual scene. Several studies show that infants rapidly learn multiple word–referent pairs by accruing statistical evidence across word–scene pairings [6,7]. Hence, a learner who is faced with multiple potential referents for a novel word on any single learning scene might store possible word–referent pairings across trials, i.e. evaluate the statistically regular co-occurrences between words and referents, and finally map individual words to their referents through this cross-trial evidence [8]. This kind of learning is shown to be sufficiently rapid and robust [6]. Indeed, humans are naturally predisposed to acquire words in their native language, a capacity that may involve the triple challenge of: (i) extracting (i.e. segmenting) and storing a word out of a continuous speech stream, (ii) inferring one or more possible referents within the visual scene and (iii) mapping the segmented word onto its referential/pragmatic meaning(s), and/or grammatical role(s) [9,10]. Linking the target word to its intended referent within the visual context is not a trivial task, as the number of potential referents in a complex scene is typically indefinite [11–13]. A final step in this process includes extending the acquired word over a potentially infinite set of appropriate referents within the same semantic category, for instance using the word ‘dog’ for dogs that the given learner has never seen before [5,14], and to an open-ended set of novel utterances [15].

A variety of cues are known to assist language learners in each of these processes involved in word learning [1,16]. Studies suggest that word frequency [17], phonotactic regularities [18–22], tactile cues [23] and prosodic modulation [24,25] orient word segmentation in infants. Importantly, carers across multiple cultures use a special speech register (infant-directed speech or ‘parentese’) characterized by elevated fundamental frequency (pitch) and intonational contours [26,27], hyperarticulated vowels ([28,29], but see recent contrasting evidence [30–32]), and high emotional content [33]. These acoustic adjustments in speech are shown to engage attention in the communication act, facilitating word segmentation for both infants [34,35] and adults [36]. In addition, a critical cue in word segmentation is the position of an item, which can also affect word acquisition. Research suggests that the general property of primacy and recency in memory abilities may focus attention on words at the edges of an utterance in infants [37–39] and adults [36,40,41]. Seidl & Johnson [38] found that infants segment words from the edges of utterances more readily than from the middle of utterances [1,39,40]. In other words, memory biases may constrain the processing of auditory input, allocating the learners' cognitive resources to the edges of the auditory utterances [42]. Although cognitive bias to the edges of utterances might be related to prosodic modifications such as initial strengthening and final lengthening of words at utterance boundaries [39], very little attention has been paid to the effect of other prosodic cues on learning words at utterance boundaries. Crucially, a number of studies suggest that statistical regularities in words guide categorization of visual objects [43–46]. Finally, as to the word–referent mapping process, research suggests that statistical patterns within words [47,48] or phrases [44,49], and the speaker's eye gaze [50] guide word–object associations in infants. In addition, much research suggests that prosody is particularly important in word–referent mapping, as it guides the infants' attention to the relevant speech component and the corresponding visual input [51], affecting their ability to acquire the intended referent [5,37,52–55]. However, in contrast to research on word segmentation and word–object fast mapping, little research has addressed perceptual cues affecting word–meaning mapping in a cross-situational task, and the extension of acquired words over novel appropriate referents [5,56,57]. A recent study by Filippi *et al*. [58] suggests that word learning is aided in adults when target words, which are consistently paired with referents across multiple visual contexts, are marked with a pitch contrast typical of infant-directed speech (IDS). Furthermore, this study suggested that pitch enhancement has a stronger effect than other possible visual and acoustic perceptual spotlights in aiding word learning in adults.

In this work, we disentangled, for the first time, the relative effect of two critical cues on word learning, prosody and position of the target words in a sentence, within a cross-situational statistical word learning task. Specifically, we tested the prediction that pitch enhancement typical of IDS and word position at the final edge of utterances significantly support word learning across multiple visual contexts, both alone and when combined.

Surprisingly, although much research has investigated the effect of perceptual salience markers in aiding word learning in infants and adults, no research we know of has addressed this effect in school-aged children. Examining subjects at this age of rapid and steady vocabulary growth is particularly relevant to enhance our understanding of the developmental curve of language learning from infancy through child- and adulthood. Critically, in this study, we tested third graders (8- to 9-year-olds) in a cross-situational word learning task. Our work provides empirical evidence suggesting that positioning a target word in utterance-final position and/or marking it with a pitch emphasis typical of IDS enables word learning in third graders. We found that the combination of these two cues significantly boosts word learning.

## 2. Material and methods

### 2.1. Participants

A total of 56 children (26 female, mean age = 8;11, range: 8–10; s.d. = 6.7 months) took part in the study. They were all monolingual native speakers of German and were recruited from an elementary school in a middle-to-high socio-economic neighbourhood in Vienna (Austria).

Exclusion criteria included bilingualism, known developmental learning disorders, and visual or auditory impairment. Participants were given colouring pens in exchange for their participation.

### 2.2. Material

In this study, we adopted the EIM (target sound string Extraction, referential category Inference and word–meaning Mapping) task developed in Filippi *et al.* [59]. This paradigm uses complex naturalistic images of target objects, providing a realistic visual parsing challenge.

**Figure 1.**
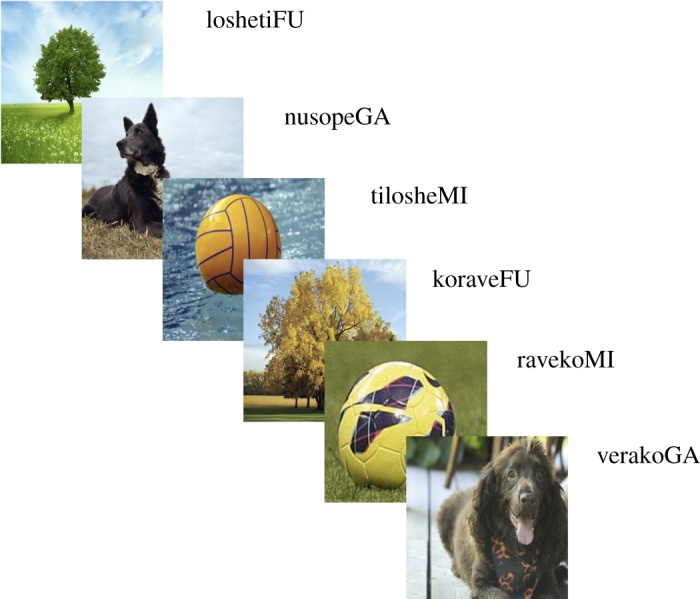
Example stimuli presentation series in the *Pitch peak* + *Utterance-final* condition. In each experimental condition, participants were exposed to 36 successive stimuli, consisting of images paired with an auditory utterance of four monosyllabic words. Each image category—dog, tree, ball—was linked only to a specific word (target word) randomly assigned to that referential category (different for different subjects). In this example, /fu/ always co-occurs with the category ‘tree’, /ga/ with ‘dog’ and /mi/ with ‘ball’ (capitalized in the figure).

The stimuli consisted of photographic images, presented on an LCD touchscreen monitor, paired with artificial language utterances presented over headphones. These spoken utterances contain a target word, which is associated with the intended image category (hereafter ‘semantic category’) of the photograph (figure 1). The co-occurrence between target words and their intended referent was the only consistent statistical cue that could help participants learn the target word–referent mapping. Participants had to identify the target words across the utterances, infer the intended semantic category from the photographs, and link these two together into a word–meaning pair which allows them to subsequently extend the acquired word to novel utterances and to new visual contexts (novel images). Custom software (Experimenter v. 3.5) written in Python v. 2.6 was used to present the stimuli and collect touchscreen responses.

#### 2.2.1. Images

Thirty-six unique full-colour images of real-life scenes were selected each depicting one of three intended visual semantic categories: ‘dog’, ‘tree’ and ‘ball’. The images were downloaded from ‘Creative Commons’ websites and scaled to 300 × 300 pixels. Care was taken that no obvious emotional or written content was depicted in these pictures.

#### 2.2.2. Sounds

We invented an artificial language made of 36 four-word utterances (hereafter ‘utterances’). Each word was a CV (consonant + vowel) monosyllabic unit. Thus, each utterance was a string of four nonsense monosyllabic ‘words’, including the target word (table 1; sound samples in electronic supplementary material). The CV syllables came from a pool of five vowels (a, e, i, o, u) and 12 consonants, namely four stops (p, t, k, g), four fricatives (f, v, s, sh) and four sonorants (m, n, l, r). Care was taken that no words or parts of utterances of our artificial language coincided with real words of the language spoken by the participants (German). As shown in table 1, in each utterance, across all conditions, each monosyllabic word occurred only once.

**Table 1.** Utterances included in our artificial language, subdivided by semantic category (dog, tree, ball) and learning condition. Target words are capitalized. Each target word was randomly assigned to a different semantic category for each subject. We adopted three different sets of target words and utterances, which were randomly assigned across participants.

	target words: ‘FU’, ‘GA’, ‘MI’							
	training				test			
conditions: *Utterance-final, Pitch peak* + *Utterance-final condition*								
semantic category A	pesonuFU	nusopeFU	sonupeFU	penusoFU	kovetiFU	tivekoFU	vetikoFU	kotiveFU
	tisheloFU	loshetiFU	shelotiFU	tilosheFU	nusheloFU	loshenuFU	shelonuFU	nulosheFU
	koveraFU	ravekoFU	verakoFU	koraveFU	rasopeFU	pesoraFU	soperaFU	rapesoFU
semantic category B	pesonuGA	nusopeGA	sonupeGA	penusoGA	kovetiGA	tivekoGA	vetikoGA	kotiveGA
	tisheloGA	loshetiGA	shelotiGA	tilosheGA	nusheloGA	loshenuGA	shelonuGA	nulosheGA
	koveraGA	ravekoGA	verakoGA	koraveGA	rasopeGA	pesoraGA	soperaGA	rapesoGA
semantic category C	pesonuMI	nusopeMI	sonupeMI	penusoMI	kovetiMI	tivekoMI	vetikoMI	kotiveMI
	tisheloMI	loshetiMI	shelotiMI	tilosheMI	nusheloMI	loshenuMI	shelonuMI	nulosheMI
	koveraMI	ravekoMI	verakoMI	koraveMI	rasopeMI	pesoraMI	soperaMI	rapesoMI
conditions: control condition, *Pitch peak condition*								
semantic category A	FUpesonu	nuFUsope	sonuFUpe	penusoFU	FUkoveti	tiFUveko	vetiFUko	kotiveFU
	FUtishelo	loFUsheti	sheloFUti	tilosheFU	FUnushelo	loFUshenu	sheloFUnu	nulosheFU
	FUkovera	raFUveko	veraFUko	koraveFU	FUrasope	peFUsora	sopeFUra	rapesoFU
semantic category B	GApesonu	nuGAsope	sonuGApe	penusoGA	GAkoveti	tiGAveko	vetiGAko	kotiveGA
	GAtishelo	loGAsheti	sheloGAti	tilosheGA	GAnushelo	loGAshenu	sheloGAnu	nulosheGA
	GAkovera	raGAveko	veraGAko	koraveGA	GArasope	peGAsora	sopeGAra	rapesoGA
semantic category C	MIpesonu	nuMIsope	sonuMIpe	penusoMI	MIkoveti	tiMIveko	vetiMIko	kotiveMI
	MItishelo	loMIsheti	sheloMIti	tilosheMI	MInushelo	loMIshenu	sheloMInu	nulosheMI
	MIkovera	raMIveko	veraMIko	koraveMI	MIrasope	peMIsora	sopeMIra	rapesoMI

	target words: ‘NA’, ‘TU’, ‘VI’							
	training				test			
conditions: *Utterance-final, Pitch peak* + *Utterance-final condition*								
semantic category A	pesomuNA	musopeNA	somupeNA	pemusoNA	kofegiNA	gifekoNA	fegikoNA	gikofeNA
	gisheloNA	loshegiNA	shelogiNA	gilosheNA	shemuloNA	loshemuNA	shelomuNA	mulosheNA
	koferaNA	rafekoNA	ferakoNA	korafeNA	rasopeNA	pesoraNA	soperaNA	rapesoNA
semantic category B	pesomuTU	musopeTU	somupeTU	pemusoTU	kofegiTU	gifekoTU	fegikoTU	gikofeTU
	gisheloTU	loshegiTU	shelogiTU	gilosheTU	shemuloTU	loshemuTU	shelomuTU	mulosheTU
	koferaTU	rafekoTU	ferakoTU	korafeTU	rasopeTU	pesoraTU	soperaTU	rapesoTU
semantic category C	pesomuVI	musopeVI	somupeVI	pemusoVI	kofegiVI	gifekoVI	fegikoVI	gikofeVI
	gisheloVI	loshegiVI	shelogiVI	gilosheVI	shemuloVI	loshemuVI	shelomuVI	mulosheVI
	koferaVI	rafekoVI	ferakoVI	korafeVI	rasopeVI	pesoraVI	soperaVI	rapesoVI
conditions: control condition, *Pitch peak condition*								
semantic category A	NApesomu	muNAsope	somuNApe	pemusoNA	NAkofegi	giNAfeko	fegiNAko	gikofeNA
	NAgishelo	loNAshegi	sheloNAgi	gilosheNA	NAshemulo	loNAshemu	sheloNAmu	mulosheNA
	NAkofera	raNAfeko	feraNAko	korafeNA	NArasope	peNAsora	sopeNAra	rapesoNA
semantic category B	TUpesomu	muTUsope	somuTUpe	pemusoTU	TUkofegi	giTUfeko	fegiTUko	gikofeTU
	TUgishelo	loTUshegi	sheloTUgi	gilosheTU	TUshemulo	loTUshemu	sheloTUmu	mulosheTU
	TUkofera	raTUfeko	feraTUko	korafeTU	TUrasope	peTUsora	sopeTUra	rapesoTU

	target words: ‘NA’, ‘TU’, ‘VI’							
	training				test			
semantic category C	VIpesomu	muVIsope	somuVIpe	pemusoVI	VIkofegi	giVIfeko	fegiVIko	gikofeVI
	VIgishelo	loVIshegi	sheloVIgi	gilosheVI	VIshemulo	loVIshemu	sheloVImu	mulosheVI
	VIkofera	raVIfeko	feraVIko	korafeVI	VIrasope	peVIsora	sopeVIra	rapesoVI

	Target words: ‘PI’, ‘RU’, ‘SA’							
	training				test			
conditions: *Utterance-final, Pitch peak* + *Utterance-final condition*								
semantic category A	gefonuPI	nufogePI	fonugePI	genufoPI	kovetiPI	tivekoPI	vetikoPI	kotivePI
	tisheloPI	loshetiPI	shelotiPI	tiloshePI	nusheloPI	loshenuPI	shelonuPI	nuloshePI
	kovemaPI	mavekoPI	vemakoPI	makovePI	mafogePI	gefomaPI	fogemaPI	magefoPI
semantic category B	gefonuRU	nufogeRU	fonugeRU	genufoRU	kovetiRU	tivekoRU	vetikoRU	kotiveRU
	tisheloRU	loshetiRU	shelotiRU	tilosheRU	nusheloRU	loshenuRU	shelonuRU	nulosheRU
	kovemaRU	mavekoRU	vemakoRU	makoveRU	mafogeRU	gefomaRU	fogemaRU	magefoRU
semantic category C	gefonuSA	nufogeSA	fonugeSA	genufoSA	kovetiSA	tivekoSA	vetikoSA	kotiveSA
	tisheloSA	loshetiSA	shelotiSA	tilosheSA	nusheloSA	loshenuSA	shelonuSA	nulosheSA
	kovemaSA	mavekoSA	vemakoSA	makoveSA	mafogeSA	gefomaSA	fogemaSA	magefoSA
conditions: control condition, *Pitch peak condition*								
semantic category A	PIgefonu	nuPIfoge	fonuPIge	genufoPI	PIkoveti	tiPIveko	vetiPIko	kotivePI
	PItishelo	loPIsheti	sheloPIti	tiloshePI	PInushelo	loPIshenu	sheloPInu	nuloshePI
	PIkovema	maPIveko	vemaPIko	makovePI	PImafoge	gePIfoma	fogePIma	magefoPI
semantic category B	RUgefonu	nuRUfoge	fonuRUge	genufoRU	RUkoveti	tiRUveko	vetiRUko	kotiveRU
	RUtishelo	loRUsheti	sheloRUti	tilosheRU	RUnushelo	loRUshenu	sheloRUnu	nulosheRU
	RUkovema	maRUveko	vemaRUko	makoveRU	RUmafoge	geRUfoma	fogeRUma	magefoRU
semantic category C	SAgefonu	nuSAfoge	fonuSAge	genufoSA	SAkoveti	tiSAveko	vetiSAko	kotiveSA
	SAtishelo	loSAsheti	sheloSAti	tilosheSA	SAnushelo	loSAshenu	sheloSAnu	nulosheSA
	SAkovema	maSAveko	vemaSAko	makoveSA	SAmafoge	geSAfoma	fogeSAma	magefoSA

The artificial language was subdivided into three different sets of 12 utterances, each of which contained the target word that referred to one of the three semantic categories depicted in the images (as in Filippi [58]). Specifically, each set of utterances shared one distinctive word, the target word, which consistently occurred in association with the corresponding visual semantic category. Hence, there were three semantic sets of utterances, i.e. sets of utterances corresponding to a specific visual semantic category (dog, tree or ball). All non-target words were systematically shared across utterances of the three semantic categories, and therefore had no consistent referential link to the visual stimuli (table 1). Within each learning condition, all non-target words were identical and systematically occurred in the same position across semantic categories. Consider, for instance, the utterance ‘loFUsheti’. The ‘lo’ and ‘sheti’ parts of the utterance occurred also within the utterances ‘loGAsheti’ and ‘loMIsheti’. Hence, only the monosyllabic words shared within each semantic set of utterances constituted target words mappable to a specific visual semantic category. Across learning conditions, within each semantic category, forward transitional probabilities between monosyllabic words, i.e. the probability of one word given the occurrence of the preceding word within each utterance, were 0, 0.25 or 0.50.

We wanted to avoid that children who were tested would reveal the correct word–meaning associations to children who still had to go through the experiment. To this goal, we adopted three different sets of target words and utterances (all built according to the description above), which were randomly assigned across participants (table 1). The three sets of target words were the following: (i) fu/ga/mi; (ii) na/tu/vi and (iii) pi/ru/sa.

In order to avoid co-articulation between adjacent words, and following Filippi *et al*. [59], each word was recorded individually. Acoustic parameters of each word were then modified using PRAAT [60]. In particular, the words' pitch and duration were modulated using the pitch-synchronous overlap add (PSOLA) algorithm [61]. PSOLA is a method based on decomposition of a signal into a series of elementary waveforms in such a way that each waveform represents one of the successive pitch periods of the signal and the sum (overlap add) of them reconstitutes the signal. The basic algorithm for the PSOLA technique consists of three steps. First, the speech waveform is divided into smaller, short-term analysis segments. Second, a mathematical function is applied to the signal, centring it on the successive instants *t*_m_, called ‘pitchmarks’. These marks are set at a pitch-synchronous rate on the voiced parts of the signal and at a constant rate on the unvoiced parts. Finally, these segments are modified, by either repeating or leaving out speech segments, depending on whether the target fundamental frequency of the signal is higher or lower than the fundamental frequency of the source signal. The remaining segments are recombined into a synthesized signal through overlapping and adding. The synthesized signal has the same spectral envelop as the source signal, but a different fundamental frequency. To change the duration of the signal, the speech segments may be repeated multiple times, to increase the duration—or eliminated, to decrease the duration. The segments are then combined into a synthesized signal using the overlap add technique.

In the Control and Utterance-final conditions (see ‘Learning conditions’ section), pitch, loudness and duration were normalized for all words within each utterance. The monosyllabic words were concatenated without pauses to form four-word utterances. Here, the target words' pitch was normalized to have the same *F*_0_ as the three other words (*M* = 210.2 Hz; s.d. = 0.22 Hz). In the Pitch peak and Pitch peak + Utterance-final conditions, loudness and duration were normalized for all words within each utterance. In these two learning conditions, in order to highlight the perceptual salience of the target word, its pitch value was elevated (*M *= 314.23 Hz; s.d. = 5.97 Hz; Max = 419.45 Hz; s.d. = 0.55 Hz). We adopted a pitch deviation of one octave, a pitch excursion typical of IDS across multiple languages [26]. Adopting a pitch exaggeration typically employed in IDS allows us to analyse the effect of highly perceptible pitch enhancement in the process of word learning. Mean intensity of target and non-target monosyllabic words was 69.97 dB (s.d. = 0.08) relative to peak amplitude. The duration of each word was normalized (*M* = 404 ms; s.d. = 3 ms).

### 2.3. Learning conditions

We adopted a between-subjects design; 14 participants were included in each of the following four learning conditions.

(1) *Control condition*: the position of the target word was varied systematically across each utterance, appearing in each of the four ‘slots’ with equal frequency. Here, the only cue that could support successful word learning would be the consistent co-occurrence of the target word with the corresponding semantic category (hereafter ‘statistical cue’). This level of information is present in all experimental conditions.
(2) *Utterance-final condition*: identical to the control condition, with the exception that the target word always occurred in the last slot in each utterance.
(3) *Pitch peak condition*: the pitch contrast manipulation described above was used in addition to the statistical cue. The position of the target word was varied systematically across each utterance, appearing in each of the four ‘slots’ with equal frequency.
(4) *Pitch peak* + *Utterance-final condition*: identical to the pitch peak condition, with the exception that the target word always occurred in the last slot in each utterance.

### 2.4. Training and testing procedure

Children were tested individually in a quiet room at their school, in a single session of approximately 15 min. Experimenters sat in the same room, at approximately 2 m from the participants.

An explicit learning paradigm was used. Participants were instructed through a cartoon-video created on *goanimate.com*. Here, a cartoon alien explained that they could participate in an ‘Alien Language Learning Game’ in which they would see a series of pictures and hear the sounds that the imaginary alien would use to describe those pictures. Participants were instructed that the experiment consisted of a training and a test phase. They were asked to do their best to understand as much as they could of this ‘alien’ language in the training, and told that their mastery of the language would be assessed in a test phase following the training. They were instructed that prior to the experiment start, they would run a practice phase in order to familiarize themselves with the ‘game’ procedure. As the actual experiment, the practice phase included training and a test phase. The practice-training consisted of six trials in which German utterances adjusted to a monotone pitch were played in association with a target image. The practice-test consisted of six trials, in which three images were shown and a monotone German utterance played. Here, children were instructed to touch the image that the sentence referred to. Two children, who did not answer correctly to more than three test trials in the practice phase, thus showing misunderstanding of the experimental procedure or lack of motivation, were excluded from the data analysis.

In both the training and the test phases of the actual experiment, the artificial language was manipulated as described in the ‘Learning conditions’ section above. Each target word was randomly assigned to a different semantic category for each subject. The training session consisted of 36 exposure trials and lasted approximately two minutes. Each utterance, and each image, was presented only once. The auditory unit–image pairs were presented in a random order across participants. For each slide, the onset of the utterance was aligned with the onset of image presentation. Consequently, only in the control and pitch peak conditions, where the target word's position was varied systematically across each utterance, onset of the target word coincided with onset of image presentation in 9 of 36 trials. In all training trials, the image remained on screen for a further 1500 ms after the end of the auditory unit's presentation.

After the training session, participants received a multiple-choice test. In the test phase, participants were presented with a novel four-word utterance, containing one of the three target words. Here, we used 12 novel utterances per semantic category, for a total of 36 novel utterances. In each test trial, one utterance was played, and three images were simultaneously shown on screen (again, one image per semantic category). Each utterance was associated once with a set of three probe images presented simultaneously, yielding 36 test trials. The onset of images presentation coincided with the onset of the auditory utterance. Participants were asked to indicate which image matched the auditory unit by touching one of the three images on the screen. The images remained on screen for 4000 ms after the end of the auditory unit's presentation. Thus, participants could thus make their choice from the end of the auditory stimulus playback to up to 4 s after the sound ended. No feedback was provided. An interval of 1000 ms followed the subject's response on each trial prior to the onset of the next trial. The order of presentation of the utterance-image trials, as well as the left-to-right arrangement of the three images on the monitor was randomized for each subject. Test utterances were the same for all participants within each learning condition. Presenting novel images probes the participants' ability to apply the acquired word to a potentially infinite set of new instances of its intended reference. The novel utterances examined their ability to identify the acquired word within an open-ended set of new utterances.

## 3. Results

Statistical analyses were performed using SPSS for Mac OS X v. 19.

**Figure 2.**
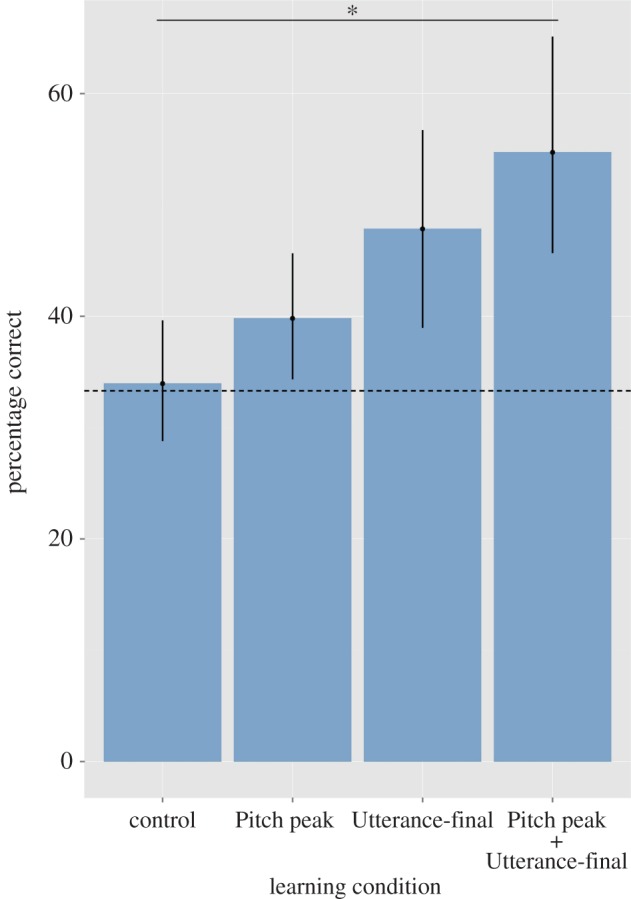
Percentage of correct responses in each experimental condition. Error bars represent 95% confidence intervals. Chance performance level is set at 33% (dashed line). All conditions except the control condition were significantly better than chance. The horizontal line (*) indicates the significant pairwise comparison between the control condition and the Pitch peak + Utterance-final condition (*p *= 0.037).

We excluded timeouts from the analyses because they could not be analysed as either correct or incorrect responses. A binomial test revealed that participants performed significantly better than chance (33.3%) in all conditions (Utterance-final and Pitch peak: *p* < 0.01, one-tailed; Pitch peak + Utterance-final condition: *p* < 0.001, one-tailed) except the control condition (*p *= 0.344, one-tailed) (figure 2). A binary logistic regression model was built within the generalized linear model framework, to compare responses across learning conditions. Data across all subjects were modelled using a binomial distribution and a logit link function. Semantic category was entered as a within-subject predictor variable and learning condition as a between-group predictor variable. The dependent variable was the proportion of correct choices in participants’ responses (where chance = 33.3%) over the number of test trials for which a response was entered. In other words, the number of trials was calculated by subtracting the number of total test trials included in our design, i.e. 12 per semantic category, minus the number of timeouts. The model revealed a significant main effect of learning condition (Wald$\chi_{3}^{2}=8.823$, *p *= 0.032), no significant effect of semantic category (Wald $\chi_{2}^{2}=4.439$, *p *= 0.109) and no significant interactions between semantic category and learning condition (Wald$\chi_{6}^{2}=6.940$, *p *= 0.326). Pairwise comparisons between the control condition and all the other learning conditions, using Bonferroni's correction, revealed a significant difference only between the control condition and the Pitch peak + Utterance-final condition (Wald $\chi_{1}^{2}=6.256$, *p *= 0.037; Cohen's *d *= 0.75; *r* = 0.35). Differences in learning performance did not reach significance between the control and the Utterance-final conditions (Wald $\chi_{1}^{2}=3.460$, *p *= 0.189; Cohen's *d *= 0.56; *r* = 0.27), and between the control and the Pitch peak conditions (Wald $\chi_{1}^{2}=1.615$, *p *= 0.611; Cohen's *d *= 0.25; *r* = 0.12) (figure 2).

## 4. Discussion

Word learning includes multiple processes: word segmentation and storage, reference induction from the visual context(s) and word–referent mapping. A number of cues and perceptual salience markers as, for instance, word position within the utterance and prosodic modulation of the signal, aid the language learners in this task. Prior studies have addressed the effect of both these cues in infants [26,35,37–39,42,52] and of pitch enhancement in adults [37,59]. However, to our knowledge little attention has been given to the role of perceptual and attentional highlight markers typical of IDS in children. This study was designed to determine the effect of words' pitch contrasts of IDS and Utterance-final position in facilitating word learning in school-age children, who are still very actively acquiring new vocabulary.

We found that 8- to 9-year-old children can acquire three new word–meaning mappings in approximately 2 min (36 exposure trials) in all conditions except the control condition (co-occurrence alone), which did not provide any perceptual or positional cues for learning. This suggests that the co-occurrence of target word and its intended referent with only one of the two salience markers we examined in this study is sufficient for word learning in school-age children. However, among the three successful conditions, only participants in the Pitch peak + Utterance-final condition reached a level of learning performance that significantly differed from the learning performance in the control condition. Hence, our data suggest that performance in this EIM task is significantly boosted when the two cues (words' pitch enhancement and Utterance-final position) are combined.

Being limited to the examination of pitch exaggeration and Utterance-final position, this study leaves open the effect of other words' prosodic marking, or of Utterance-initial position. We may speculate that further cues aiding memory, for instance placing the target word in Utterance-initial position—if combined with cross-situational co-occurrence of target word and visual semantic category and with pitch marking of the target word—would ease word learning in school-age children.

Our findings are in line with other studies [36,38,39,49], suggesting that edge alignment facilitates word learning in preverbal infants. Our work extends these studies, suggesting that recency effects alone may not be sufficient to ease word segmentation, and that prosodic modifications of the words in Utterance-final position are necessary to facilitate word learning. Our results are particularly interesting if linked to findings described in Filippi *et al.* [59] on adults, where an identical experimental paradigm was adopted. Filippi *et al.* [59] suggested that the word–reference co-occurrence cue alone is sufficient for word learning in adults. In addition, the authors found that word learning is boosted only when pitch enhancement marks the target word, also when the position of target words varied across utterances. A possible explanation for the discrepancy between the present research and Filippi *et al.* [59] may be due to differences in adults' and children's sensitivity to salient features in the spoken utterances or to different cognitive resources for word learning in children as compared to adults [55]. It can be argued that, in contrast to word learning in adults, word learning in school-age children is significantly boosted when multiple cues and/or salient perceptual modifications mark target words. In fact, our results complement previous findings on word learning, showing that processing multiple sources of information (e.g. unfamiliar, but statistically regular phonetic variations and word–objects associations) at the same time supports cross-modal learning [46,62,63]. Our data suggest that for school-age children, the combination of *specific* cues aiding memory and enhancing word perception—namely cross-situational statistical regularities between acoustic signal and visual image, pitch enhancement and utterance-final position—facilitate word learning. More work will be needed to explore the combination of other types of cues enhancing the target word (e.g. word lengthening, or vowel hyperarticulation) with word position in utterances within a cross-situational word learning task. Crucially, further work disentangling the role of statistical regularities within the utterances and the relative contribution of other learning cues is suggested.

In addition, future research should aim at improving the experimental design making the task easier for children, possibly testing also younger participants on the same task. This may be achieved, for instance, by implementing the experiment into a videogame paradigm—thus making the task more involving for children [64,65], by including more acoustic variation, or by elongating the training phase. This work specifically focuses on word learning, which we define as including generalizations of target words used in previously unheard utterances, and applied to novel images. Further work is required to establish whether children would succeed in recognizing the target words in a simpler test task, where, for instance the same images used in the training are paired with novel utterances. This line of research will contribute to develop a full picture of factors aiding all the different processes and mechanisms involved in language learning across different stages of the language development curve. Furthermore, future research comparing children's and adults' performance in language learning tasks should examine whether children are more sensitive than adults to word position, perhaps using an artificial language where word position and/or prosodic marking is linked to syntactic properties in the signal.

Our results extend research concerning the beneficial effect of perceptual marking for tasks that engage memory and attentional resources, as was the case for our word learning task [1,5,44,45,51,54]. Moreover, the present study contributes additional evidence suggesting that 8- to 9-year-old language learners exploit pitch enhancement and word Utterance-final position as cues that co-occur with the intended target words and visual categories across learning contexts. These findings align with previous research on natural languages, underlining the importance of positional regularities for language acquisition and processing [1].

This research may be relevant for educational programmes aiming to facilitate the process of second language(s) learning, or to investigate the specific uses of voice modulation as a teaching strategy in school-age children. Further research addressing the effect of word markers in enhancing children's language learning will improve our understanding of the processes underlying language development and acquisition.

## Supplementary Material

Table 1

## Data accessibility

Data are available from the Dryad Digital Repository (http://dx.doi.org/10.5061/dryad.gh31g) [66].
