# Supplementary material for: Utterance-final position and pitch marking aid word learning in school-age children
Source: R Soc Open Sci. 2017 Aug 16;4(8):161035. doi: 10.1098/rsos.161035 (PMC5579076; doi:10.1098/rsos.161035)
Supplement: Table 1 [file rsos161035supp1.pdf]

Target words: „FU“, „GA“, „MI“

Conditions: *Utterance-final, Pitch peak + Utterance-final condition*

	Training				Test			
semantic category A	pesonuFU	nusopeFU	sonupeFU	penusoFU	kovetiFU	tivekoFU	vetikoFU	kotiveFU
	tisheloFU	loshetiFU	shelotiFU	tilosheFU	nusheloFU	loshenuFU	shelonuFU	nulosheFU
	koveraFU	ravekoFU	verakoFU	koraveFU	rasopeFU	pesoraFU	soperaFU	rapesoFU
semantic category B	pesonuGA	nusopeGA	sonupeGA	penusoGA	kovetiGA	tivekoGA	vetikoGA	kotiveGA
	tisheloGA	loshetiGA	shelotiGA	tilosheGA	nusheloGA	loshenuGA	shelonuGA	nulosheGA
	koveraGA	ravekoGA	verakoGA	koraveGA	rasopeGA	pesoraGA	soperaGA	rapesoGA
semantic category C	pesonuMI	nusopeMI	sonupeMI	penusoMI	kovetiMI	tivekoMI	vetikoMI	kotiveMI
	tisheloMI	loshetiMI	shelotiMI	tilosheMI	nusheloMI	loshenuMI	shelonuMI	nulosheMI
	koveraMI	ravekoMI	verakoMI	koraveMI	rasopeMI	pesoraMI	soperaMI	rapesoMI

Conditions: Control condition, *Pitch peak condition*

	Training				Test			
semantic category A	FUpesonu	nuFUsope	sonuFUpe	penusoFU	FUkoveti	tiFUveko	vetiFUko	kotiveFU
	FUtishelo	loFUsheti	sheloFUti	tilosheFU	FUnushelo	loFUshenu	sheloFUnu	nulosheFU
	FUkovera	raFUveko	veraFUko	koraveFU	FUrasope	peFUsora	sopeFUra	rapesoFU
semantic category B	GApesonu	nuGAsope	sonuGApe	penusoGA	GAkoveti	tiGAveko	vetiGAko	kotiveGA
	GAtishelo	loGAsheti	sheloGAti	tilosheGA	GAnushelo	loGAshenu	sheloGAnu	nulosheGA
	GAkovera	raGAveko	veraGAko	koraveGA	GArasope	peGAsora	sopeGAra	rapesoGA
semantic category C	MIpesonu	nuMIsope	sonuMIpe	penusoMI	MIkoveti	tiMIveko	vetiMIko	kotiveMI
	MItishelo	loMIsheti	sheloMIti	tilosheMI	MInushelo	loMIshenu	sheloMInu	nulosheMI
	MIkovera	raMIveko	veraMIko	koraveMI	MIrasope	peMIsora	sopeMIra	rapesoMI

Target words: „NA“, „TU“, „VI“

Conditions: *Utterance-final, Pitch peak + Utterance-final condition*

	Training				Test			
semantic category A	pesomuNA	musopeNA	somupeNA	pemusoNA	kofegiNA	gifekoNA	fegikoNA	gikofeNA
	gisheloNA	loshegiNA	shelogiNA	gilosheNA	shemuloNA	loshemuNA	shelomuNA	mulosheNA
	koferaNA	rafekoNA	ferakoNA	korafeNA	rasopeNA	pesoraNA	soperaNA	rapesoNA
semantic category B	pesomuTU	musopeTU	somupeTU	pemusoTU	kofegiTU	gifekoTU	fegikoTU	gikofeTU
	gisheloTU	loshegiTU	shelogiTU	gilosheTU	shemuloTU	loshemuTU	shelomuTU	mulosheTU
	koferaTU	rafekoTU	ferakoTU	korafeTU	rasopeTU	pesoraTU	soperaTU	rapesoTU
semantic category C	pesomuVI	musopeVI	somupeVI	pemusoVI	kofegiVI	gifekoVI	fegikoVI	gikofeVI
	gisheloVI	loshegiVI	shelogiVI	gilosheVI	shemuloVI	loshemuVI	shelomuVI	mulosheVI
	koferaVI	rafekoVI	ferakoVI	korafeVI	rasopeVI	pesoraVI	soperaVI	rapesoVI

Conditions: Control condition, *Pitch peak condition*

	Training				Test			
semantic category A	NApesomu	muNAsope	somuNApe	pemusoNA	NAkofegi	giNAfeko	fegiNAko	gikofeNA
	NAgishelo	loNAshegi	sheloNAgi	gilosheNA	NAshemulo	loNAshemu	sheloNAmu	mulosheNA
	NAkofera	raNAfeko	feraNAko	korafeNA	NArasope	peNAsora	sopeNAra	rapesoNA
semantic category B	TUpesomu	muTUsope	somuTUpe	pemusoTU	TUkofegi	giTUfeko	fegiTUko	gikofeTU
	TUgishelo	loTUshegi	sheloTUgi	gilosheTU	TUshemulo	loTUshemu	sheloTUmu	mulosheTU
	TUkofera	raTUfeko	feraTUko	korafeTU	TUrasope	peTUsora	sopeTUra	rapesoTU
semantic category C	VIpesomu	muVIsope	somuVIpe	pemusoVI	VIkofegi	giVIfeko	fegiVIko	gikofeVI
	VIgishelo	loVIshegi	sheloVIgi	gilosheVI	VIshemulo	loVIshemu	sheloVImu	mulosheVI
	VIkofera	raVIfeko	feraVIko	korafeVI	VIrasope	peVIsora	sopeVIra	rapesoVI

Target words: „PI“, „RU“, „SA“

Conditions: *Utterance-final, Pitch peak + Utterance-final condition*

	Training				Test			
semantic category A	gefonuPI	nufogePI	fonugePI	genufoPI	kovetiPI	tivekoPI	vetikoPI	kotivePI
	tisheloPI	loshetiPI	shelotiPI	tiloshePI	nusheloPI	loshenuPI	shelonuPI	nuloshePI
	kovemaPI	mavekoPI	vemakoPI	makovePI	mafogePI	gefomaPI	fogemaPI	magefoPI
semantic category B	gefonuRU	nufogeRU	fonugeRU	genufoRU	kovetiRU	tivekoRU	vetikoRU	kotiveRU
	tisheloRU	loshetiRU	shelotiRU	tilosheRU	nusheloRU	loshenuRU	shelonuRU	nulosheRU
	kovemaRU	mavekoRU	vemakoRU	makoveRU	mafogeRU	gefomaRU	fogemaRU	magefoRU
semantic category C	gefonuSA	nufogeSA	fonugeSA	genufoSA	kovetiSA	tivekoSA	vetikoSA	kotiveSA
	tisheloSA	loshetiSA	shelotiSA	tilosheSA	nusheloSA	loshenuSA	shelonuSA	nulosheSA
	kovemaSA	mavekoSA	vemakoSA	makoveSA	mafogeSA	gefomaSA	fogemaSA	magefoSA

Conditions: Control condition, *Pitch peak condition*

	Training				Test			
semantic category A	PIgefonu	nuPIfoge	fonuPIge	genufoPI	PIkoveti	tiPIveko	vetiPIko	kotivePI
	PItishelo	loPIsheti	sheloPIti	tiloshePI	PInushelo	loPIshenu	sheloPInu	nuloshePI
	PIkovema	maPIveko	vemaPIko	makovePI	PImafoge	gePIfoma	fogePIma	magefoPI
semantic category B	RUgefonu	nuRUfoge	fonuRUge	genufoRU	RUkoveti	tiRUveko	vetiRUko	kotiveRU
	RUtishelo	loRUsheti	sheloRUti	tilosheRU	RUnushelo	loRUshenu	sheloRUnu	nulosheRU
	RUkovema	maRUveko	vemaRUko	makoveRU	RUmafoge	geRUfoma	fogeRUma	magefoRU
semantic category C	SAgefonu	nuSAfoge	fonuSAge	genufoSA	SAkoveti	tiSAveko	vetiSAko	kotiveSA
	SAtishelo	loSAsheti	sheloSAti	tilosheSA	SAnushelo	loSAshenu	sheloSAnu	nulosheSA
	SAkovema	maSAveko	vemaSAko	makoveSA	SAmafoge	geSAfoma	fogeSAma	magefoSA
